# Understanding Students’ Mental Well-Being Challenges on a University Campus: Interview Study

**DOI:** 10.2196/15962

**Published:** 2020-03-05

**Authors:** Sun Young Park, Nazanin Andalibi, Yikai Zou, Siddhant Ambulkar, Jina Huh-Yoo

**Affiliations:** 1 School of Information University of Michigan Ann Arbor, MI United States; 2 School of Medicine Northwestern University Evanston, IL United States; 3 College of Computing and Informatics Drexel University Philadelphia, PA United States

**Keywords:** emerging adults, university students, life events, mental wellbeing, mental wellness, mental health, social support

## Abstract

**Background:**

Research shows that emerging adults face numerous stressors as they transition from adolescence to adulthood. This paper investigates university students’ lived experiences of maintaining mental well-being during major life events and challenges associated with this transitional period. As we continue to design health technology to support students’ mental health needs, it is imperative to understand the fundamental needs and issues particular to this phase of their life to effectively engage and lower the barriers to seeking help.

**Objective:**

This study first aimed to understand how university students currently seek and receive support to maintain their mental well-being while going through frequent life events during this period of emerging adulthood. The study then aimed to provide design requirements for how social and technical systems should support the students’ mental well-being maintenance practice.

**Methods:**

Semistructured interviews with 19 students, including graduate and undergraduate students, were conducted at a large university in the Midwest in the United States.

**Results:**

This study’s findings identified three key needs: students (1) need to receive help that aligns with the perceived severity of the problem caused by a life event, (2) have to continuously rebuild relationships with support givers because of frequent life events, and (3) negotiate tensions between the need to disclose and the stigma associated with disclosure. The study also identified three key factors related to maintaining mental well-being: time, audience, and disclosure.

**Conclusions:**

On the basis of this study’s empirical findings, we discuss how and when help should be delivered through technology to better address university students’ needs for maintaining their mental well-being, and we argue for reconceptualizing seeking and receiving help as a colearning process.

## Introduction

### Background

Emerging adulthood, a transitional period between adolescence and adulthood, is characterized by various challenges individuals struggle with while taking on adult roles and responsibilities [1]. The literature defines emerging adults as those aged from 18 to 29 years [1]. Some key characteristics of emerging adulthood are instability, identity exploration, self-focus, and feeling in-between (ie, not quite an adult and not quite an adolescent) [1]. These characteristics are particularly prevalent in students on college campuses as they face increased academic pressures in addition to sudden shifts in their social, geographic, and economic life contexts [2]. According to a recent national survey of college students in the United States [3], over the last year, 85% of the students felt overwhelmed by their given tasks, and 49.8% of the students reported feeling hopeless.

During this transitional phase of emerging adulthood, university students experience various life events at different scales, ranging from entering college, leaving home and moving to a new city, and making new networks, to taking finals and looking for a job [4]. These life events become potential stressors as students are expected to increase their personal responsibility and independence and make constant adjustments, including personal, emotional, and social adjustments [5]. Research shows that support from peers, family, and health care resources aids their life satisfaction, self-esteem, perceived social acceptance, and well-being [6,7].

With advances in technology, researchers have approached the problem of supporting students’ mental health through a variety of technological interventions and tools. Novel tools and mobile apps have been developed to support clinical interventions, risk predictions, and self-monitoring of illness triggers and symptoms, such as home clinical therapy mobile app [8], self-monitoring tools that help track and reduce stress [9], and social media platforms to provide social support and monitor mental health risks [10] and suicidal ideation [11]. These tools help to monitor and forecast mental illness and allow clinical therapies and treatments to be more accessible. However, such clinical approaches to technological interventions could leave out those who do not consider their problems to be mental illness or disorder problems but a “normal” part of maintaining mental well-being [12].

We build upon recent work in human-computer interaction (HCI) and Health Informatics [13-17], which approaches university students’ struggles and challenges as a mental well-being problem rather than as a clinical, mental illness problem. This approach helps address the problems that students need to engage but often ignore because they consider those problems not to be mental health concerns but “normal” problems that occur during emerging adulthood [12]. Focusing on mental well-being helps us understand students’ everyday lived experiences of muddling through major life events during their emerging adulthood.

With this perspective, this study first aimed to understand how university students currently seek and receive support to maintain their mental well-being while going through frequent life events during their emerging adulthood. The study then aimed to provide design requirements for how social and technical systems should support the students’ mental well-being maintenance practice. In this paper, we report findings from semistructured interviews with 19 students on a university campus. We identified three key needs: (1) the need to receive help that matches the perceived severity of a problem caused by life events, (2) the need to constantly draw on new support givers because of frequent life events, and (3) the need to negotiate the tension between the benefits of self-disclosure and the stigma associated with such disclosure. On the basis of this study’s findings and analysis, we make the following contributions:

- We extend our knowledge about university students’ understandings and perceptions of mental health and coping strategies.
- We discuss how students strive to maintain their mental well-being and perceive the boundary between what counts as mental “illness” vs “wellness” in assessing whether to get help.
- We present design implications for sociotechnical systems to support the needs of students to better maneuver through this important transitional life phase.

### Related Work

The American Psychological Association defines emerging adulthood as the “in-between age,” referring to the phase of development between adolescence and adulthood [18]. Regardless of socioeconomic prospects, those between the ages of 18 and 29 years commonly struggle with “feeling in between” [19]. Emerging adults, particularly those who attend universities, go through a great amount of stress. According to the US Department of Education (2011), student enrollment in college increased to 38% since 1999, reaching its peak. However, the majority of those who drop out of college do so in their first year [20], indicating that the transition process is crucial to students’ success. In addition to dealing with academic pressure, they are expected to make a series of adjustments, ranging from academic assimilation to personal (eg, changed interests), emotional (eg, home sickness), and social (eg, finding new friends) adjustments [5].

What makes emerging adulthood more uniquely challenging than other periods is the intensity of multiple life events mounting up together in a short period. The danger of multiple life events occurring in a short period has been noted in life events research—critical life events (eg, moving, starting a new job, leaving family, and paying new rent or mortgage) and their accumulation can predict long-term medical and psychiatric illness problems [21,22]. The major life events of emerging adults often begin with entering college and leaving home and their old, existing networks. To help such students with the struggles brought by the transitional phase of emerging adulthood, the American College Health Association has identified coping with stress as a high priority issue in the Healthy Campus initiative [23]. This initiative takes a multifaceted approach to mental well-being, which includes environmental (eg, managing air quality), physical (eg, eating well), and social (eg, student clubs) health. Universities offer Counseling and Psychological Services (CAPS) with mental health professionals. CAPS further provide campus-wide programs for first-generation college students and underserved students to reduce attrition (eg, drop out) [24-26]. Despite the benefit of these initiatives, the literature still notes the problems of students not utilizing CAPS until emergency situations occur, if they ever do [27]. Nearly 80% of the students who die by suicide never participate in counseling services [28-30]. The reasons for this underutilization include lack of awareness [31], stigma [32], long waiting list times [33], and preference for self-management [34].

Besides the institutional and national effort to support students, many studies showed that students mostly seek and receive social support from their existing networks in dealing with their struggles. Peer and family support plays a critical role in offering students a sense of belonging [7], affirmation of their concerns [35], encouragement and empathy [36], and facilitation of mental health maintenance as a compensatory measure when other support is unavailable [37,38]. Social support enabled by technology, such as social network sites and Web-based and virtual social communities, has shown the potential benefits to supporting students, such as facilitating students’ identity transitions [39], offering access to help when lacking in-person support [40], addressing unpleasant experiences with negative content on the Web [41], and resolving stigmatized concerns [42,43]. However, research indicates that barriers related to seeking and getting social support, including stigma of mental illness [44,45], a sense of autonomy (eg, “I don’t need a doctor to solve this problem”), a fear of losing one’s positive image (ie, “face”) [46], and secrecy and negative cultural attitudes toward mental illness [47], often suppress help-seeking behaviors [48], which hampers students from getting timely and proactive support. These previous studies on emerging adulthood highlight both the critical need for proper support for university students during their stressful life transitional period and the associated barriers that hinder students from seeking and receiving such support.

Researchers developed and evaluated technological interventions for mental health issues such as anxiety and depression. Some examples include Harmony (a mobile health app that assists patients, caregivers, and clinicians in managing serious illnesses) [49], DStress (a mobile app that coaches users in balancing compliance and achievement toward daily goals of stress reduction) [9], the UPRIGHT project (in which researchers designed and validated resilience-based interventions for promoting early adolescents’ mental health) [6], and Intellicare (a suite of skill-focused apps that successfully provide users with more flexibility in choosing a suitable treatment) [15]. Furthermore, technology-mediated therapies have been implemented (a conversational mobile app, Pocket Skills, that provides Dialectical Behavioral Therapy [8] and Web-based cognitive behavioral therapy) [50]. Although these interventions have shown their effectiveness and usefulness for treating mental illness, they mainly focus on those who are clinically diagnosed and actively seeking treatment, not those who do not consider their problems as serious mental health conditions but approach them by attempting to maintain their mental well-being.

Upon reviewing the literature on emerging adults’ mental health on college campuses, we find examining how students engage everyday life challenges and the everyday maintenance aspect of their mental well-being to be a critical, emerging, and underinvestigated research topic in HCI and Health Informatics communities. As we continue to design health technology to support students’ mental health needs, it is imperative to understand the fundamental needs and issues particular to this phase of their life to effectively engage and lower the barriers to seeking help. Building on the previous work summarized here, this study includes the following research questions (RQs):

- RQ1: How do university students perceive their problems and maintain their mental well-being while going through various, frequent life events?
- RQ2: What are the implications for designing sociotechnical systems that are tailored to meeting university students’ unique needs around life events?

## Methods

We conducted interviews with 19 university students (6 undergraduate and 13 graduate) from a large US Midwest university for a semistructured interview study. The inclusion criteria were as follows: (1) English speaking, (2) interested in speaking about their mental health or well-being, and (3) enrolled either as an undergraduate or a graduate student of the university. We recruited the participants through school-wide departmental email lists and word of mouth, and we stopped data collection when saturation was reached.

The study was approved by the Institutional Review Board (IRB) of the first author’s university, and all coauthors who had access to the transcripts were registered as study team members. Our interview sessions were conducted in private rooms on the university campus. A member of the research team began the interview by informing the participants about the study’s goals and interview procedures and assuring them that they could skip any questions they did not feel comfortable answering or stop the interview at any point. The participants were told that confidentiality would be maintained and that they could withdraw their participation at any time without any consequence. This study followed IRB guidelines, which state that if any crisis situation was observed (eg, threats and suicidal posts), the participant would be immediately referred to the staff at the University CAPS. Each participant received US $15 for study participation.

The duration of each interview session ranged from approximately 1 to 1.5 hours. The questions were designed to help us gain further insights that might not be uncovered in public reports on overall campus climate, where we see increases in student struggles with stress, anxiety, depression, and requests for on-campus counseling services. The interview questions probed the students’ process of identifying their stressors, their experiences of seeking and receiving support or help, their development and adaptation of coping strategies to maintain mental health or well-being, and the tools and technologies they used in this process. At the end of the interview, the researchers offered information about college counseling services and other mental health support resources available on campus to those who were in need or had no previous experience of them.

All of the interviews were audio recorded and transcribed for the analysis. This study followed all of our institution’s confidentiality, privacy, and security policies for handling and storing all the participants’ data. Three researchers analyzed the data using the open coding method [51], using the Atlas.ti 7 (ATLAS.ti Scientific Software Development GmbH) qualitative coding software program. We conducted axial coding to explore and develop the key attributes and dimensions of each theme particular to the RQs. Working collaboratively, we explored, compared, and revised the themes as the analysis progressed. We revised the themes through a series of discussions and iterations until consensus was reached. We also conducted affinity diagramming [52] to generate common themes and identified any breakdowns and design implications during the analysis process. We followed the Contextual Inquiry Design process [52] to identify common themes from the affinity diagram to generate design requirements.

## Results

### Overview

In this section, we first provide demographic information on this study’s participants and then present the three needs that were identified in this study. These three needs are critical to understanding how students maneuver through various life events, seek help, and maintain their mental health and well-being.

### Study Participants

Table 1 shows the overall participant demographics. The students ranged in age between 20 and 29 years (mean 25 years, SD 4 years), and 13 students were female, and 6 students were male. In all, 7 students were international students. Reported ethnicities were as follows: East Asian (6), non-Hispanic white (6), Indian (5), and African American (2). A total of 14 out of the 19 students had had experiences with professional counseling services. Two (P7 and P19) students were clinically diagnosed with a psychological disorder.

**Table 1:** Demographic information about our study participants.

Participant number	Age (years)	Gender	Ethnicity	Counseling and psychological services, or professional service experience
P1	28	Male	Chinese	Yes
P2	24	Male	Korean	Yes
P3	30	Female	Taiwanese	Yes
P4	20	Female	Indian	Yes
P5	21	Female	White	No
P6	29	Male	White	Yes
P7	20	Female	White	Yes
P8	29	Male	Indian	Yes
P9	20	Male	Indian	Yes
P10	27	Female	Taiwanese	Yes
P11	23	Female	White	No
P12	20	Male	Indian	No
P13	24	Female	White	Yes
P14	24	Female	Indian	No
P15	25	Female	White	Yes
P16	25	Female	Chinese	Yes
P17	30	Female	Korea	Yes
P18	25	Female	African American	No
P19	28	Female	Middle Eastern	Yes

### The Need to Receive Help That Aligns With the Severity of the Problem

The participants emphasized the importance of how and when they receive help for their problems based on how they perceived the severity and duration of the problem they were struggling with. We found that knowing or not knowing when a problem would end and having multiple stressful events accumulate at once mainly affected students’ perception about the severity of their problems. This perceived severity then affected the students’ expectations about help seeking. Below, we describe how life events affected perceived problem severity and what students considered “good help” to be as a result.

#### The Severity of the Problem Is Perceived Differently Based on Life Events

The problems students struggled with in maintaining their mental well-being included adjusting to new cities and life on new campuses, meeting new people, paying for tuition, examinations, and job searches. Some of these problems involved more certainty in terms of how long they would last, such as final exams; others, on the other hand, seemed to last forever, such as job search–related stress. Accordingly, uncertainty regarding how long their problem would last affected how students perceived the severity of the problem and how they approached it. For instance, until receiving her recent job offer, P3, who was an international graduate student, had been “really, really stressed.” She explained the following:

The past year, when I was first year [of the graduate school], I was really stressed... I would never go back to my home [in China] during the time because I can only come back with a job offer.

Here, P3 described the stressful event as having lasted for a year, which significantly affected her mental well-being during that year. She did not previously know, nor could she have predicted, how long the job search would take while it was happening. The job search was stressful, and the fact that it occurred over an indefinite period of time added a considerably increased amount stress. P3 was expressing the severity of the problem in relation to the event duration because she was unable to start or plan other things until her job status was secured, directly affecting her legal status to live in the United States after graduation.

In perceiving the severity of their problem, we found that a few students even put a predefined time boundary on the problem (how long it would last) and remarked that their current problems would pass and there would eventually be closure. This perception helped them get through the problem. For instance, when P3 was having a problem with her boyfriend during her job search period, she mentioned that she had given herself 3 months (a limited amount of time) to deal with the stress and move on when she would start a new job:

I told myself just three months to get over it. I was eating snacks, watching TV shows, and doing exercises.

P3

Starting college as an independent young adult brought multiple problems in school, work, and relationships at the same time. These multiple problems occurring in the same period exacerbated the perceived severity of each individual problem. For instance, P5 experienced a high level of stress when she had a serious problem with her boyfriend while also applying for summer internships and working on her final exams. P8, similarly, shared an extreme, distressing moment because of the stress brought on by multiple, urgent work responsibilities:

There was this one time when I had a very large amount of work to do. I had a part-time job, had one big [class] assignment, and had one [class] project due, all within the next 48 hours. Even if I stayed up the entire 48 hours, there was no way I was going to finish everything. I knew that because of the amount of work involved, and that was really causing me stress because either the assignment, the project or the part-time job had to suffer, and I wasn’t sure which. I was being paid for the part-time job, the project was a group project and if I don’t do the work then nobody gets a grade, and the assignment was an individual assignment for me, but it was a large part of the grade, so I had to leave at least one.

P8

P8 further explained that he had to seek emotional support from family members and friends and go to his academic advisor for useful advice. As in the case of P8, multiple problems could occur during the same transitional period, thus requiring the student to navigate the different types of support resources available to receive the proper help. This indicates that merely having one therapist may not work well for students.

#### Seeking Help That Is Accessible and Available at an Appropriate Time

The urgency and timing of seeking help varied together with whether the students framed the problem as having ended or as ongoing. All students in the study commented on the importance of receiving support during the particular period when the struggles were still happening to effectively deal with their struggles. However, ensuring timely support was challenging because seeking support required a great amount of effort in their hectic daily lives. For instance, students mentioned that they often found resource constraints in their existing support environments on campus. Several participants mentioned that existing support resources such as CAPS and other professional services sometimes failed to address their need to receive help at the right time, especially for time-pressured and serious concerns. This was because of tedious nature of scheduling phone calls, long wait times for in-person appointments, inflexible working hours, and an inefficient screening process, all of which made it difficult to access care in a timely manner:

There was a pretty long waiting time. The initial session was I think after a week and a half, and then the next appointment was after a couple of weeks. Time is the only problem [with CAPS], and I realize that the timing is not in their control because of the number of people here.

P8

Another student described his experience:

I decided to go to CAPS, and was kind of disappointed because at first I filled out that whole questionnaire and the first meeting wasn’t even an actual meeting it was just going over the questionnaire with a counseling student.

P9

These factors that delayed the timing of receiving help frequently made the students uninterested in reaching out to CAPS for help.

As their perception of the problem severity was time bound, when students were unable to find proper help in a timely fashion, they tried to manage it on their own or consider that the problem might go away some time after they endured that particular stressful event. We found that after a certain amount of time passed, the students did not necessarily want to be reminded of an issue, nor did they feel the need for professional help anymore. Rather, they wanted to put their problems behind them, letting them resolve on their own over time or trying to ignore them. Ensuring help at the appropriate time was particularly tricky for students who were required to change their locations periodically:

I came back home for the summer so I couldn’t see them [therapists at CAPS] again. I could have come in the fall but it wouldn’t have been the same. It would a long break and I would have thought through some things on my own and stuff like that. I didn’t go to them afterwards.

P9

As the participants, including P9, left the campus for summer and winter breaks for various reasons such as internships, vacations, or other duties, many had to leave in the middle of getting help without fulfilling their needs, and they felt that the help that came later was meaningless.

In addition, we found that when seeking and receiving help, some students experienced a discrepancy between what they expected and how the help was actually delivered. Types of problems they had might be similar to those of their peers, as emerging adults on campus were going through similar life events. Although the perceived severity might differ, students felt that their struggles might not look as serious to others as they perceived them to be. In fact, they experienced difficulty in getting proper recognition or serious consideration from others. Consequently, students quickly lost interest or motivation to actively seek help when they did not feel they were getting enough recognition or empathy from others upon initial sharing. P3 mentioned how her problem was not appropriately acknowledged or addressed by the therapists’ consultation:

The therapist said, ‘Ok it’s been one hour, I think we are going to wrap up.’ Then I was so surprised, what!? Are we finished? I thought you were going to give me some suggestions. At least some homework for what I am going to do... Then, she [therapist] said, ‘no, no you have so many stress to relieve, so this time you just relieve all your tension, you just let your sore come out. And next time we will talk about others.’

P3

In this quote, P3 showed her frustration about how her severe problem was mostly treated as one of the many other stresses without the proper understanding she expected. In this study, this kind of experience, not getting sufficient recognition of the severity of their problems, made even those students who initially sought help consider that their problems might not be serious enough for professional treatment and that they might need to be manage the problem themselves.

Many of the students in the study wanted to receive help in a more casual way through frequent and informal conversations, instead of fixed (regular but limited) and formal conversations. As most students saw their problems as issues that could come and go and get better or worse, and as similar to what their peers were all dealing with, rather than as mental health issues requiring formal diagnoses, they preferred casual and frequent interactions, which allowed them easy access when needed. P15 expressed the need to see mental health practitioners more frequently and informally as a preventative approach, rather than seeing them only when “something was going wrong,” such as “when feeling depressed, when feeling anxious, when having maniac episodes whatever it might be.”

Similarly, a couple of students proactively managed their stress through frequent hangouts with friends. P14 shared her experience of using her regular get together as an outlet for her distress:

It makes me feel good about how things are. I get to offload my emotional stress. I do that [talk about things in mind/head] once a week when we all get together. But on a daily basis we don’t talk as much about anything emotional or anything about our job, just like, have you had food, where are you going, things like that.

P14

P14 designated a weekly time to “talk about things” with a small group of friends who she hung out with on a daily basis as a way to focus on sharing stressful issues, as they might go through similar stressful life events and share similar struggles. Having weekly gatherings with close friends made sharing problems comfortable instead of awkward or uncomfortable.

In sum, students struggled with various problems brought by life events, especially multiple problems concurrently happening during a transitional period, and they tried to look for help that could effectively address the problems. Despite the seriousness of a problem and the critical need for help, the problems students struggled with were mostly considered to be part of normal emerging adulthood–related issues, not “serious” ones, by the students themselves and even by others whom they tried to ask for help. Students thus preferred casual help, or they took a preventative approach so that those “normal” problems would not develop into “serious” ones.

### The Need to Build, Rebuild, and Maintain Support Givers for Frequent Life Events

All students participating in the study had recently gone through major life events and changes: they had left their hometowns or relocated, sought or made new friends in college or at work, or made changes in their career—for example, switching majors, changing schools, delaying their degree, and coming back to school from industry. These transitions caused changes in social support structures, exacerbating stress and problems in maintaining their mental well-being. The following quote from P8 illustrates the highly stressful period of transitions and the loss and regaining of social support:

The transition time was very rough because I was used to a place with a lot of people and a lot of activities. When I first came here... I don’t know, I found a lot of simple things to be very difficult. I didn’t have my own mode of transportation, so had to rely on the bus services. Small experiences like that really added to my stress levels and I found the fact I lived away from the main campus, [which] meant that I had to spend a lot of time on my own, which I didn’t like because I like hanging around people. When I was staying alone it was quite difficult for me.

P8

#### Relationship Building Is Constantly Needed to Have Access to Potential Support Givers

The various life events the participants experienced not only forced them to quickly adjust to new life contexts and rebuild their social connections but also made it difficult to maintain their existing social connections. For this reason, students often felt that the support they wanted was not readily available or workable, but inconsistent; supportive social groups or support givers were likely to come and go. For instance, P18 described her premed group members with whom she initially developed deep relationships, and she described how these relationships were enhanced by their common struggles and active support for each other. Later, she felt helpless and discouraged when all the members of the group transferred to other universities or decided to pursue something else:

I think with pre-med courses, it’s nice to have a community where you can be stressed out together and fall on each other for advice and study together and help each other. But then when everybody dropped, and it was just me, I think that’s when it got to be very stressful because I was doing everything on my own.

P18

The short-term and inconsistent social support received from their existing support givers was somewhat detrimental to the students’ mental health, often resulting in sudden feelings of being lost or alone. Although some relationships were strong and long lasting, the students needed to be prepared to lose connections and relationships they often put a large amount of time and effort into building and rebuilding.

Most of the students in this study experienced the diminishment, disappearance, or replacement of intimate friends they previously relied on to receive support, such as the replacement of high school friends with a small group of close friends from the freshman cohort, or they experienced the quality of relationships with old friends changing over time. P9 explained how the friendships he had with close friends growing up and their support for each other became rather unstable and shifted after 3 years of college as they experienced conflict and disagreement:

These were probably the two people I would go to if I had trouble with school and things, but after I had a disagreement with them, I felt very alone and isolated. Now, I’m a better friend to them but I think because we had that break we’re not as close as we used to be. It’s always in the back of my mind that we had this fight so I’m not that close to them as much anymore.

P9

This shows that even with existing support givers (eg, intimate friends), effort was still needed to maintain relationships as all parties went through different stages and events of their own lives during emerging adulthood, affecting their current or future situations, viewpoints, and needs.

While going through different life events and needing to seek appropriate help, the students encountered situations where they needed to determine how to disclose and share their problems with the new social connections they had just built or developed. The new social connections were usually people they had recently met in classes, at work, at various events, or where they lived. For instance, P10 shared her experience of sharing her concerns with a new friend she had started to get close with recently:

I talk a lot this semester with Olivia [her classmate], but like until the end of the semester, not really earlier. I feel like this semester, the early part of this semester I’m always alone because I am mostly in Fishbowl to finish 520 assignment or something. (...) She also shares her concern as well. I think for the academic part, we are very similar. In the same situation, I guess. And we haven’t got internship yet and we’re also worried about finding a project, final exams, so it’s hard. Our schedules are more similar. We sometimes work together on weekends.

P10

Having similar situations, interests, or struggles as other individuals helped students quickly build new relationships and potentially share their problems, although the sharing might initially be limited to their common interests.

While sharing problems with newly developed social connections, students did not share their real, deep problems upfront, unless those were the commonly shared issues they already knew about each other (eg, met at a counseling session). They wanted to share problems in a friendship after building a relationship and worried that sharing such issues in the beginning of the relationship could lead to making some “unattractive” or “undesirable” impressions:

I wouldn’t necessarily want people to know that to be the first thing they know about me. I want them to know me as a person. I feel like, yes, that’s an important thing to know about me in a friendship and stuff, but I don’t want that to be the first thing.

P13

#### Seeking Alternatives to Support Givers on the Web

Owing to the challenges of having a constant change of support givers while going through frequent life events, the students often sought alternative forms of support, such as making new Web-based social connections and asking for help (eg, Facebook groups, mental health–related web forums, and anonymous others on Reddit or Instagram). As Web-based support is always accessible, students found that this served as a support baseline that they could contact regularly or whenever they wanted to check in as a casual (not as structured and serious as a CAPS or professional service) but also steady (with minimum effort to set up) way of maintaining their mental well-being. However, despite the benefits of having Web-based help, students reported issues with the sustainability and the consistency of support based on their experiences. They believed that the size of the group, the qualities of the anonymous members, and whether there was guidance and a moderator within the Web-based communities often affected the quality of the support they received. Many, including P19, mentioned the double-edged sword aspect of anonymity in Web-based groups and communities regarding the anonymous members and expected behaviors:

I don't like Reddit, not at all! 'Cause Reddit is totally about anonymity. And I don't enjoy that. I think anonymity is to some degree dehumanizing. It's really unaccountable. And people allow their worst selves out because they don't understand impact when they're anonymous.

P19

According to the participants, the size of the group mattered for the perceived quality of group dynamics and conversation. When the Web-based support group was too large, the conversation among the group members would become disorganized and derailed by new information, which often made participants feel less valued. In contrast, with small groups, the students often felt they did not get enough diverse opinions, which could lead to a lack of effective solutions or ideas to address their problems. Nearly all of the participants advocated 7 to 10 members as an ideal group size for sharing their concerns and having effective group discussions:

Less than 10 participants [would be good] in order to have sufficient dialogue and participation among everyone.

P4

The personal qualities of the web members were also seen as an important consideration to ensure that the support was sustainable. Students considered being faithful, committed, and respectful of others’ privacy the most needed qualities of support members as potential support givers, especially given the relative anonymity of many support spaces. The students also commented on their desire to engage both intimate friends and strangers in their envisioned Web-based support group to receive the most useful help, as each provided different types of support on different subjects. In addition, moderated interactions within support groups were seen as critical for students to better receive quality social support. They remarked that careful moderating and monitoring helped maintain the support environment as a healthy and useful place for everyone to get consistent support for the long term. A few students (P8, P12, P14, and P16) who had never used any Web-based support group expressed their interest in joining a small group conversation moderated by professionals who could understand the members, properly react to what was said in the group, and maintain group positivity:

You have a different mental state when you’re talking to a therapist compared to talking to just anonymous people. I might not be able to respond if one [a person] was sad or depressed. In a sense, a therapist response would be much nicer and much more understanding compared to what I can provide.

P14

Similarly, some who already had experience with moderated Web-based support groups also preferred to have a more structured support environment where a moderator provided group therapy or assigned particular goal-oriented tasks to help members achieve their goals. Several advocated for having more than one moderator or professional monitor to track the conversations to ensure that the members would interact appropriately, particularly in anonymous environments.

### The Need to Negotiate Tensions Between the Benefit of Disclosure and the Stigma Associated With Disclosure

The last challenge that students mentioned was balancing self-disclosure to seek help and the stigma that prevents such disclosure. To express a need for help, the students attempted to find ways that allowed them to avoid the stigma associated with the disclosure while also maintaining their mental well-being.

#### Students Hold Tension in Maintaining Desirable Presentations of Self

One common concern related to transitions across frequent life events, particular to emerging adulthood, was maintaining and developing a desirable presentation of self in social interactions. Although wanting to maintain a “positive” image or reputation is a well-known phenomenon in social science research [53], it becomes particularly challenging when one’s network keeps changing, as was the case for the students participating in this study. Students commented that the stigma associated with sharing personal problems could hinder the development of positive social relationships, often discouraging them from openly sharing their concerns with their social connections, such as friends, family, and the people with whom they had recently developed relationships. Particularly, as many students considered their problems might be trivial and common, they considered that what they were going through should not be overestimated as a serious problem. This thinking process aggravated their stressful situations.

The transitions during emerging adulthood made it difficult to keep up with their original support givers (ie, old and intimate friends), and it forced them to build new connections (ie, new friends). The new social connections students built were the ones with whom they wanted to further develop intimacy. These new connections were critical to their emerging adulthood in that they were the students’ most preferred source of support. Thus, to further develop these new relationships in the ways they desired, students did not want to disclose their problems too much, but they preferred to maintain a positive self-presentation toward their new connections. For example, some students wanted to maintain an image of being fun and outgoing. They worried that if they disclosed too much about their depression, sadness, or struggles, their new friends would view them differently and categorize them as “not a fun friend to be around” or “problematic.” P2 described hiding his concerns from friends in an effort to maintain the character of being fun and happy:

You have five friends and they expect you to be very fun and outgoing, you can’t help but stay in that character. It’s very difficult or you feel uncertain that if you were to reveal a little bit [of your] personal self, would they still be the same towards you know, [the] friendship or not.

P2

As in the case of P2 above, many students worried that the negative, long-term impact of sharing their concerns with their social connections, particularly with newer ones, would outweigh the potential benefits, which ultimately prevented them from getting effective help.

Owing to the tension between their need to share with friends and the associated stigma, many students put a lot of effort into sharing their problems in ways they perceived to be less risky. They would slowly and carefully bring their concerns into the conversation or look for the right time to bring up an issue. For example, we saw that some students waited to share their concerns until a friend approached them first, instead of asking for help directly; others tended to hide their true feelings, pretending to live and act in a way that conformed to how their friends viewed them, until they felt more comfortable talking about their problems. P6 explained how long it took him to feel comfortable sharing his struggles with others, explaining that there was a certain time at which such disclosure would be allowed normatively:

At the end of April when everyone is getting ready to [graduate] and started to pull out their hearts and essentially had a very close talk. That was with everyone else, and I was just sitting there and said I’m not graduating, they are, so I should at least tell them something that has been on my mind for the past few years. There was that instance where I felt should tell them.

P6

Here, the fact that his friends were all graduating and would not be around anymore made it easier for P6 to disclose the issues that he was holding onto. His friends’ transitions also made it less burdensome to deal with their reactions, which was why he chose to disclose at that time.

Disclosure was difficult when students experienced pressure to follow and meet societal expectations or standards, such as being independent financially, accomplished educationally or professionally, or settled at a culturally expected age. This finding was more apparent among the Asian students, who felt pressured to be “good” or “mature” for their families in their particular culture. For instance, P10 who started her graduate study in her late 20s explained her struggle with feeling stigmatized by her age and financial situation:

I actually borrowed money from my parents. At my age I shouldn't do that, and especially, my parents used their retire money to support me. That's a big issue. After graduation I will definitely have to find a good job and pay back. My family invested [in] me to go abroad, so that means I will have the responsibility to support my family back as well. I think that's a concern I have. (...) The other thing is my age, I would say. I think relationship and marriage is also very...not super stressful, but when I think about it, I will kind of worried about that. Because I've never had a relationship before and right now I'm almost 30. Especially my parents worry about that. I feel everything is related to my family. If my family worry about this then I will worry about that too. I know they want me to have a family in close future, but so far in my situation I don't think I have enough time to do that, seeking one and building a relationship...”

P10

Like P10, even though students talked to their parents every day to share their thoughts, we found that many of them tried not to reveal struggles related to their schoolwork because they wanted to present themselves as a smart, hardworking daughter or son.

#### Exploring a Casual, Nuanced Way of Sharing Problems

As a way to deal with the stigma associated with sharing problems, students favored communicating with their social connections (potential support givers) through images. Image-based communication included any images, such as the students’ own drawings, famous paintings, landscapes, body parts, any artifacts from their daily lives, funny or abstract pictures, and funny memes, shared through mobile or Web-based messages or social network sites. This was popular among the participants, especially for when they just wanted a small amount of casual support that could cheer them up in their daily lives or for when they wanted to provide support for someone else, without anyone explicitly seeking help:

I use Facebook and Instagram basically just to see what people are up to and for funny videos and photos of dogs and videos of dogs. I like memes a lot, so if I see a funny meme I just like it. (...) so like funny pictures and funny comments, internet memes. If I see one, I’ll just click like so that my friends will see it, and my friends will start laughing about it. I use that as a stress buster really.

P16

Similarly, sharing dog and cat photos on the Web was also a popular form of support during less energized moods.

Image-based communication allowed the students to indirectly express their nuanced feelings, as they could use some of the affordance images offer to express and share their feelings in a very subtle and subjective way, without directly revealing their true feelings or intentions. It also enabled students to adjust their own moods while sharing or posting images as a way to overcome negative feelings or curate their own feelings. Students (P11 and P16) used their social media as a digital diary, using images to share and record their moods:

I think this semester I post a lot less, but usually I post a lot. Facebook is almost like one kind of my diary. I post everything, [including] not good [ones]. Especially not good things on that. (...) I don’t know [the motivation], but I think it’s just like, letting it go, away for me to like expressing my anger or depression. Because I feel, if I cannot find a person to talk, because on Facebook it’s also a way for me to let it go and let the others know that I’m not good.

P11

Like P11, other participants expected image sharing and posting to enable them to feel more comfortable and embedded during their everyday lives, while reducing the stress and stigma related to their support-seeking behaviors. By using a platform that people use to communicate about everyday situations, the shared problems are portrayed as mundane and normal problems of daily life rather than as serious mental health problems.

## Discussion

### Overview

University students go through a critical transitional life phase of emerging adulthood between adolescence and young adulthood. This life phase requires increased responsibility, maturity, and independence, causing more stress and problems with maintaining mental well-being. For this reason, there have been increased mental health incidents on college campuses [13,54], and more researchers have been exploring technological interventions for emerging adults on university campuses [50]. Below, we describe the maintenance work required for students’ mental well-being using the literature on self-disclosure [55]. We also discuss the boundary students perceive between mental well-being and illness, which played a critical role in students seeking help. We then discuss implications for potential sociotechnical systems that can support the needs of students in better maneuvering through this important transitional phase of life.

### Negotiating Time, Audience, and Disclosure for Mental Well-Being Maintenance Work Across Life Events

This study revealed that students’ mental well-being needs constantly shift and evolve as they go through the life events associated with emerging adulthood. These transitions, shaped by life events, critically impact their mental well-being. This study’s findings draw attention to how students negotiate the three needs in their lived experiences related to time, audience, and disclosure. The severity and duration of the life events (*time*) defined the expectations of how help should be delivered. Students encountered new social relationships, which they had to constantly rebuild and maintain as sources of support. These relationships at the same time served as the *audience* for whom the students had to balance the tension between the stigma and *disclosure* of problems and seeking help. As such, these three needs are closely interconnected and constantly negotiated to maintain their mental well-being. This interconnected relationship has been identified in the studies about disclosure in HCI and Communication fields.

For instance, “time” has been found to be a critical component in when, how, and whether disclosures occur, exploring the facilitation and hindrance of disclosures [55,56]. Reasons for delayed disclosure [56,57], a delay between a significant, stressful event and the time of disclosure, include fear that the disclosure will be poorly received and not supported by others as well as moving on from the event and not feeling the need to share or reopen wounds [56]. Although delayed disclosures have primarily been studied in the context of abusive and traumatic situations (eg, sexual abuse) [58], this study shows that delayed disclosure also happens when students wait for the right “timing” to disclose their problems and seek help. An example from this study’s findings showed that one student (P5) waited until his friends’ graduation to reveal his issue to reduce the burden of having to manage friends’ reactions to his disclosure. Moreover, this was during a moment when the friends had mutually agreed to share intimate experiences as a way to reflect on their college years. The reciprocity norm of mutual self-disclosure in dyadic relationships [59] explains this observation of mutual sharing at the right time.

“Audience” is another important factor in explaining why people disclose or do not disclose stigmatizing experiences (eg, depression) in the Disclosure Decision-Making Frameworks [55,56]. The concept of audience connects with our findings on rebuilding relationships, such as the new social connections students need to build. According to Goffman, “audience” refers to the individuals for whom one performs and who might be the recipients of one’s direct or indirect disclosures as part of a help request [53]. The disclosing person assesses how these individuals, that is, the audience, might react and respond to the disclosure or lack thereof and makes a (non)disclosure decision accordingly. In this study, such an assessment includes balancing the potential benefits of disclosure with the risk of losing the status of being a fun friend to be around, especially when the goal is to rebuild and maintain new relationships with the audience. This negotiation is fraught as these new relationships entail potential disclosure to audiences who can be a source of support.

Furthermore, this study also showed that as a result of the risk of stigma associated with disclosing problems, students used fun and positively valued social media sharing to release stress for themselves and others. Previous work has argued that social media design should facilitate the disclosure of stressful situations directly or indirectly [56]. This study added to this conversation by arguing that sharing positive content is not necessarily shallow or “just for fun”; it can, in fact, be part of an activity that releases tension and stress among individuals.

### Understanding the Perceived Boundary Between Mental Wellness and Illness as a Collective Effort

In this study’s findings, students perceived most of their problems to be either normal or on a continuum between normal and serious. The latter would imply the need for seeking immediate help from a professional source, compared with the former, where the problem may resolve on its own as time passes. Students view the problem as fluctuating day to day, or even hour by hour, and as being influenced by many things in their daily lives, just like physical health. They think normal problems can progress to become serious and serious problems can revert to being normal as time passes (after a certain stressful life event is over). Coupled with stigma and barriers to self-disclosure [55], students attempted to avoid as much as possible the need to label their problems as illness problems.

The students in this study tended to consider their problems within the realm of wellness—a state where they think they have abilities to cope with the normal stresses of life and manage on their own. However, students also worried that their wellness problems could potentially develop into more serious ones and become illness problems. To prevent this progression, students actively and proactively tried to seek effective, workable resolutions. This study’s findings showed that students set a maximum time (eg, three months and two weeks) in which to resolve problems (eg, relationship breakup and being stressed about grades or job search) to prevent the problems from overwhelming their overall well-being. Having a social network to share their problems with, whereby students could set up regular weekly hangouts with close friends to prevent the problems from progressing into more serious ones helped.

In this study, we reported different perspectives between the mental health literature and findings in terms of when a problem is considered as part of “wellness” vs “illness.” This difference makes it difficult to determine when and how we should intervene to support students’ help-seeking behaviors effectively. The traditional mental health literature on emerging adulthood problematizes the students’ perceived, self-endorsed boundaries between wellness and illness, defining it as a barrier to mental health care and support [60]. The literature legitimizes self-management activities for mental health but only to a certain extent. Self-management activities, referring to seeking help from family and friends and going to meditation groups, are largely described as barriers to mental health care access. Rickwood et al [61,62] found that participants preferred to talk to friends and family rather than health care professionals, which might prevent access to health care when added to participants’ lack of emotional competence and negative attitudes toward seeking professional help. Biddle et al [63] showed that participants continuously self-assessed whether they could accommodate high levels of distress and avoid seeking help.

The health care stance on interventions is that the earlier the intervention, the better. However, students are not inclined to seek professional care easily or early on [64]. As shown in this study’s findings, students did not feel the need to continue existing professional help or start seeing a doctor or therapist because of their strong tendency to perceive their problems as normal. This tendency aligns with recent studies that show individual beliefs and perceptions, such as newly acquired independence and autonomy [65-67], as stronger factors in whether emerging adults will seek mental health support [65,68]. This study’s finding highlights the urgent need to not only better understand university students’ individualized perceptions but also help them self-reflect and re-evaluate their own perceived boundaries of where illness begins so that they can receive the support they need—whether from their social network or through professional care.

Where the boundary between wellness (ie, whether a problem is a normal part of emerging adulthood) and illness (ie, a symptom of a serious mental health disorder) lies or whether the boundary should even exist remains an open question. This might be an applicable question for the general population, such as those with mental health issues in the workplace [69], but it is the salient case for students as they are especially close to their peers who go through similar types of problems and life events during their emerging adulthood, which can make the students more ignorant or blunt about their problems. The question about the boundary has often been unclear even for health professionals because of individualized, situational factors [70]. Health care workers need to individually assess how much the unstable, uncertain experiences of a student should be treated as a healthy part of self-development and how much should be treated as a symptom of a serious mental health disorder [70]. This nuanced boundary as well as the differing viewpoints between health care professionals and students can add to maintaining student mental well-being negatively. This problem forces students on college campuses to be tolerant of severe problems and to ignore the need to get help.

We argue that this boundary between wellness and illness needs to be carefully negotiated, evaluated, and coordinated for each individual student through collective efforts among the social systems made up of responsible stakeholders: university students themselves, health care professionals, family, friends, educators, designers, institutions, and the public health sector.

### Reconceptualizing a Support Group as a Place for Colearning

In examining the ways in which students received support, including from their groups of intimate friends, Web-based groups, and social media, we noticed that being part of a “support group” had a negative connotation for our students. Many students avoided labeling the wanted help as a “support group,” as the term problematizes what should be a normal process of being supportive of each other in a seamless way throughout everyday contexts. The negative connotation associated with support groups served as a barrier to students accessing helpful resources, such as CAPS or related peer groups, on campus. It is worth investigating how to engage and assist students with various stressors in their emerging adulthood more effectively.

Accordingly, this study calls for an urgent need to reconceptualize the existing notion of support as a colearning space where individuals learn to maintain their own mental wellness: how to be aware of it, how to keep it healthy, and how to prevent potential risks. This knowledge is not something normally gained from formal education, although it should be. Importantly, in this reconceptualization, each individual can be a help giver as well as a receiver. This sort of mutual exchange has been discussed in the literature as the helper therapy principle [71], which is the central concept for building support groups. In this study, a few of those who wrote blogs or made images related to mental wellness awareness expressed their interest in working with counseling services to help their peers, based on their own experiences. At any given time, one can be a helper for multiple people on different issues, while seeking help for one’s own mental well-being maintenance work. This multiparticipatory nature of a support exchange can facilitate accountability among the helpers and the individuals receiving help.

### Design Implications

We discussed university students’ challenges and efforts to maintain their mental well-being around time, disclosure related to stigma, and the right audience. This study indicated that it is important to facilitate the negotiation of students’ own perceived boundaries of where illness begins through self-reflection and evaluation so that they can receive appropriate help. To that end, ideal sociotechnical systems should be designed in more inclusive ways that allow self-reflection to support students in managing their mental health in a casual, informal, and positive manner, rather than approaching the challenge from a mental illness perspective.

To our knowledge, only a few existing technical interventions support students’ mental health from the mental well-being maintenance perspective [14,17,72]. These systems exist either for tracking students’ mood and emotions or encouraging social support. For example, Mind Tracker [14], a tracking tool kit, uses a journal, graphic chart, and tangible modality-like clay. Its main function is to engage users in the process of emotional data collection and self-reflection in everyday life. Using Mind Tracker, the study found that having an expressive tool was beneficial to express and track students’ emotions and that this tool also served as a self-healing tool by providing a moment of mindfulness, rather than as an automatic sensing tool to detect negative emotions. In addition, Buddy [17] is a social media–based support system that offers Bell features, where users can indicate posts that they think need more careful attention than others, Special Feeds that curate those posts requiring more attention (instead of popular or trending posts) and Hug Bots that automatically deliver hugs when creating a post (instead of a Like button that often creates competition). This Buddy system was designed to help identify students who need more attention and encourage students to share their struggles with others, particularly those who need more social support in adjusting to college life.

This study’s findings show value in integrating functionality from such systems to support students’ mental well-being maintenance work. They call for investigating how existing systems and applications for the general population can be extended and designed to offer self-reflective and self-evaluative mechanisms for students’ mental well-being. For instance, existing practices, such as Expressive Art Therapy (EAT), which offers individuals the opportunity to use any expressive and creative art form (eg, singing, poetry, art, dance, or performance) to reflect on and identify what the root of a problem is, increase self-awareness, enhance social relationships, and foster emotional growth and development [73-75]. EAT can then be integrated into existing everyday systems and applications to provide nonclinical solutions. Specifically, existing media applications for self-expression, such as video blogs, amateur music sharing, sharing creative artwork or images, drawing, or poetry sharing forums, can be used to support these varieties of forms of expression as a form of EAT. Similarly, doodling can be used to benefit the process of mindfulness training with some guidance (eg, “Choose whether to document a current happy moment or think back to a happy time and doodle your memory”) [76]. Integrating such expressive capabilities into existing applications could enable students to be mindful and aware of their mental health status. In addition, integrating such an approach with Positive Psychology [6,77,78] can help students release their stress and discover positive moments and strengths, instead of focusing on the problems and the negativities. Examples include enabling the platform to suggest recording a thankful moment from the day through a photo, writing, or drawing and sharing it with friends on Instagram or in a group chat or cocreating humorous internet memes with friends on Sketch as a positive mood booster. These activities can foster emotional growth, enhance social relationships, and improve self-awareness [22,35]. These outcomes will eventually help young adults define their own perceived boundary of mental health and receive proper help when needed.

Upon developing the platform, we argue that it should be designed to facilitate the involvement of a community of all relevant stakeholders in supporting the mental well-being maintenance work of an emerging adult on campus (eg, students, health care providers, practitioners, institutions, designers, and app developers). This platform will not only help students to self-reflect and evaluate their mental well-being maintenance activities but also help stakeholders to easily analyze functionalities, use cases, and user populations of existing applications. It will also help them analyze new applications as they are developed and suggest potential connections to existing therapeutic practices of mental well-being. The platform could show usage trends of user populations, along with new features that these applications might be able to provide. It could also offer a database of EAT curated by health care professionals. The stakeholders could then exchange feedback regarding how these existing and newer applications could work together to support therapeutic mechanisms according to the life events of an individual.

The findings also indicate that it is critical to support the process of navigating and renegotiating time, audience, and disclosure. The aforementioned platform could provide a variety of approaches to requesting as well as receiving situated help, ranging from the immediate and direct to the more casual and abstract. On the basis of the mood or level of activity of a user or a particular life event, a platform could directly intervene by matching or suggesting appropriate helpers or by nudging and casually connecting with family and friends to encourage students to share their struggle with others. This could help with providing support within the appropriate time window when the impact of that event is still active. For instance, the findings showed students did not want to be reminded of their problem after a certain amount of time had passed after the issue occurred. Such a platform could also pick up on and identify potential emotional breakdowns or challenges that might come up, based on their behavioral and emotional patterns. In addition, in terms of suggesting an audience, selecting helpers from social group contacts or outsourcing highly customized help based on the topic or similar interests and needs (eg, nearby peer groups, mentors, and professional consultants) could be done for the user. Matching with others based on similar needs or interests was found to be helpful in previous work using others’ life transition trajectories as a form of social support among peer patients [79]. The incentive for anyone to become a helper could be established by keeping them informed of both the short-term and long-term positive outcomes of addressing a problem through feedback, thus alleviating tension related to stigma and facilitating the rebuilding and maintenance of relationships between users and new and potential helpers.

### Conclusions

We investigated the mental well-being maintenance practices of emerging adults on a university campus: how students perceive problems, set expectations for help, and go through the work of from whom, when, and how to seek help. We identified three needs that are required for students to maintain their mental well-being: (1) getting help at an appropriate time and in an appropriate form that aligns with the severity of the problem, (2) building relationships with potential support givers, and (3) negotiating tension between disclosure and the associated stigma. This formative study informed how sociotechnical system support for this population should be sensitive to how help is framed and offered to make it easier to embrace. We also discussed how and when help should be delivered through technology and argued for reconceptualizing help as a colearning process. This research was conducted with a subsample of emerging adults, focusing on those at a large university in the Midwest. Although the participants do not represent the broader population of emerging adults nor all university students, this study’s findings demonstrate a unique set of needs and challenges that should be acknowledged and supported in the population to which these findings theoretically generalize. This study contributes to helping mental health practitioners, researchers, and system designers understand and support students’ mental well-being maintenance practices as part of the normal course of life beyond the clinical context.

## Abbreviations

CAPS: Counseling and Psychological Services
EAT: Expressive Art Therapy
HCI: human-computer interaction
IRB: Institutional Review Board
RQ: research question

## References

[1] Arnett JJ, Žukauskienė R, Sugimura K (2014). The new life stage of emerging adulthood at ages 18-29 years: implications for mental health. Lancet Psychiatry.

[2] Eagan K, Stolzenberg EB, Ramirez JJ, Aragon MC, Suchard MR, Hurtado S (2014). The American Freshman: National Norms Fall 2014.

[3] American College Health Association (2018). American College Health Association (ACHA).

[4] Compas BE, Wagner BM, Slavin LA, Vannatta K (1986). A prospective study of life events, social support, and psychological symptomatology during the transition from high school to college. Am J Community Psychol.

[5] Hiester M, Nordstrom A, Swenson LM (2009). Stability and change in parental attachment and adjustment outcomes during the first semester transition to college life. J Coll Stud Dev.

[6] Gabrielli S, Las Hayas C, Hjemdal O, Ledertoug M, Guðmundsdóttir DG, Ólafsdóttir AS, Zwiefka A, Fullaondo A, on behalf of the UPRIGHT consortium (2018). The UPRIGHT Project: Designing and Validating Resilience-Based Interventions for Promoting Mental Wellbeing in Early Adolescence. Proceedings of the 12th EAI International Conference on Pervasive Computing Technologies for Healthcare.

[7] Hoffman M, Richmond J, Morrow J, Salomone K (2002). Investigating 'sense of belonging' in first-year college students. J Coll Stud Ret.

[8] Schroeder J, Wilkes C, Rowan K, Toledo A, Paradiso A, Czerwinski M, Gloria M, Linehan MM (2018). Pocket Skills: A Conversational Mobile Web App To Support Dialectical Behavioral Therapy. Proceedings of the 2018 CHI Conference on Human Factors in Computing Systems.

[9] Konrad A, Bellotti VM, Crenshaw N, Tucker S, Nelson L, Du H, Pirolli PL, Whittaker S (2015). Finding the Adaptive Sweet Spot: Balancing Compliance and Achievement in Automated Stress Reduction. Proceedings of the SIGCHI Conference on Human Factors in Computing Systems.

[10] Deane FP, Wilson CJ, Ciarrochi J (2001). Suicidal ideation and help-negation: Not just hopelessness or prior help. J Clin Psychol.

[11] De Choudhury M, Kiciman E, Dredze M, Coppersmith G, Kumar M (2016). Discovering Shifts to Suicidal Ideation from Mental Health Content in Social Media. Proceedings of the 2016 CHI Conference on Human Factors in Computing Systems.

[12] Arnett JJ (2001). Adolescents’ responses to cigarette advertisements for five 'Youth Brands' and one 'Adult Brand'. J Res Adolesc.

[13] DiGeronimo TF, Kadison R (2004). College of the Overwhelmed: The Campus Mental Health Crisis and What to Do About It.

[14] Lee K, Hong H (2017). Designing for Self-Tracking of Emotion and Experience with Tangible Modality. Proceedings of the 2017 Conference on Designing Interactive Systems.

[15] Mohr DC, Tomasino KN, Lattie EG, Palac HL, Kwasny MJ, Weingardt K, Karr CJ, Kaiser SM, Rossom RC, Bardsley LR, Caccamo L, Stiles-Shields C, Schueller SM (2017). IntelliCare: an eclectic, skills-based app suite for the treatment of depression and anxiety. J Med Internet Res.

[16] Park SY (2018). Social Support Mosaic: Understanding Mental Health Management Practice on College Campus. Proceedings of the 2018 Designing Interactive Systems Conference.

[17] Wohn DY, Ahmadi M, Gong L, Kulkarni I, Poojari A (2017). Designing a Social Support System for College Adjustment and Social Support. Proceedings of the Eleventh International AAAI Conference on Web and Social Media.

[18] Munsey C (2006). American Psychological Association.

[19] Arnett JJ (2000). Emerging adulthood. A theory of development from the late teens through the twenties. Am Psychol.

[20] Rausch JL, Hamilton MW (2006). Goals and distractions: explanations of early attrition from traditional university freshmen. Qual Rep.

[21] Dohrenwend BS, Dohrenwend BP, Dohrenwend BS, Dohrenwend BP (1974). A brief historical introduction to research on stressful life events. Stressful Life Events: Their Nature and Effects.

[22] Paykel E (2001). The evolution of life events research in psychiatry. J Affect Disord.

[23] (1979). American College Health Association. J Am Coll Health Assoc.

[24] Bragg DD, Kim E, Barnett EA (2006). Creating access and success: academic pathways reaching underserved students. New Dir Commun Coll.

[25] Deil-Amen R (2011). Socio-academic integrative moments: rethinking academic and social integration among two-year college students in career-related programs. J High Educ.

[26] Inkelas KK, Daver ZE, Vogt KE, Leonard JB (2007). Living–learning programs and first-generation college students’ academic and social transition to college. Res High Educ.

[27] Erdur-Baker O, Aberson CL, Barrow JC, Draper MR (2006). Nature and severity of college students' psychological concerns: a comparison of clinical and nonclinical national samples. Prof Psychol Res Pr.

[28] Drum DJ, Brownson C, Denmark AB, Smith SE (2009). New data on the nature of suicidal crises in college students: shifting the paradigm. Prof Psychol Res Pr.

[29] 27 RPG (2006). National Survey of Counseling Center Directors. American College Counseling Association.

[30] Kisch J, Leino EV, Silverman MM (2005). Aspects of suicidal behavior, depression, and treatment in college students: results from the spring 2000 national college health assessment survey. Suicide Life Threat Behav.

[31] Westefeld JS, Homaifar B, Spotts J, Furr S, Range L, Werth JL (2005). Perceptions concerning college student suicide: data from four universities. Suicide Life Threat Behav.

[32] The Carter Center (2003). The Carter Center.

[33] O'Malley K, Wheeler I, Murphey J, O'Connell J (1990). Changes in levels of psychopathology being treated at college and university counseling centers. J Coll Stud Dev.

[34] Czyz EK, Horwitz AG, Eisenberg D, Kramer A, King CA (2013). Self-reported barriers to professional help seeking among college students at elevated risk for suicide. J Am Coll Health.

[35] Meadows GN, Burgess PM (2009). Perceived need for mental health care: findings from the 2007 Australian Survey of Mental Health and Wellbeing. Aust N Z J Psychiatry.

[36] Repper J, Carter T (2011). A review of the literature on peer support in mental health services. J Ment Health.

[37] O'Leary K, Bhattacharya A, Munson SA, Wobbrock JO, Pratt W (2017). Design Opportunities for Mental Health Peer Support Technologies. Proceedings of the 2017 ACM Conference on Computer Supported Cooperative Work and Social Computing.

[38] Pettit JW, Roberts RE, Lewinsohn PM, Seeley JR, Yaroslavsky I (2011). Developmental relations between perceived social support and depressive symptoms through emerging adulthood: blood is thicker than water. J Fam Psychol.

[39] Morioka T, Ellison NB, Brown M (2016). Identity Work on Social Media Sites: Disadvantaged Students' College Transition Processes. Proceedings of the 19th ACM Conference on Computer-Supported Cooperative Work & Social Computing.

[40] Konijn EA, Utz S, Tanis M, Barnes SB (2008). Transformed social interaction in mediated - interpersonal communication. Mediated Interpersonal Communication.

[41] Li G, Xiaomu Z, Tun L, Jiang Y, Ning G (2016). SunForum: Understanding Depression in a Chinese Online Community. Proceedings of the 19th ACM Conference on Computer-Supported Cooperative Work & Social Computing.

[42] Rains SA, Wright KB (2016). Social support and computer-mediated communication: a state-of-the-art review and agenda for future research. Ann Int Commun Assoc.

[43] Wright KB, Bell SB, Wright KB, Bell SB (2003). Health-related support groups on the internet: linking empirical findings to social support and computer-mediated communication theory. J Health Psychol.

[44] Conner KO, Koeske G, Brown C (2009). Racial differences in attitudes toward professional mental health treatment: the mediating effect of stigma. J Gerontol Soc Work.

[45] Corrigan P (2004). How stigma interferes with mental health care. Am Psychol.

[46] Goffman E (1963). Stigma: Notes on the Management of Spoiled Identity.

[47] Park S, Kim I, Lee SW, Yoo J, Jeong B, Cha M (2015). Manifestation of Depression and Loneliness on Social Networks: A Case Study of Young Adults on Facebook. Proceedings of the 18th ACM Conference on Computer Supported Cooperative Work & Social Computing.

[48] Eisenberg D, Downs MF, Golberstein E, Zivin K (2009). Stigma and help seeking for mental health among college students. Med Care Res Rev.

[49] Tuli A, Singh P, Sood M, Deb KS, Jain S, Jain A, Wason M, Chadda R, Verma R (2016). Harmony: Close Knitted mHealth Assistance for Patients, Caregivers and Doctors for Managing SMIs. Proceedings of the 2016 ACM International Joint Conference on Pervasive and Ubiquitous Computing: Adjunct.

[50] Davies EB, Morriss R, Glazebrook C (2014). Computer-delivered and web-based interventions to improve depression, anxiety, and psychological well-being of university students: a systematic review and meta-analysis. J Med Internet Res.

[51] Strauss A, Corbin JM (1998). Basics of Qualitative Research: Techniques and Procedures for Developing Grounded Theory.

[52] Beyer H, Holtzblatt K (1999). Contextual design. Interact.

[53] Goffman E (1959). The Presentation of Self in Everyday Life.

[54] Hunt J, Eisenberg D (2010). Mental health problems and help-seeking behavior among college students. J Adolesc Health.

[55] Greene K, Derlega VJ, Mathews A, Vangelisti AL, Perlman D (2006). Self-disclosure in personal relationships. The Cambridge Handbook of Personal Relationships.

[56] Andalibi N, Forte A (2018). Announcing Pregnancy Loss on Facebook: A Decision-Making Framework for Stigmatized Disclosures on Identified Social Network Sites. Proceedings of the 2018 CHI Conference on Human Factors in Computing Systems.

[57] Semaan B, Britton LM, Dosono B (2017). Military Masculinity and the Travails of Transitioning: Disclosure in Social Media. Proceedings of the 2017 ACM Conference on Computer Supported Cooperative Work and Social Computing.

[58] Somer E, Szwarcberg S (2001). Variables in delayed disclosure of childhood sexual abuse. Am J Orthopsychiatry.

[59] Derlega VJ, Harris MS, Chaikin AL (1973). Self-disclosure reciprocity, liking and the deviant. J Exp Soc Psychol.

[60] Gulliver A, Griffiths KM, Christensen H (2010). Perceived barriers and facilitators to mental health help-seeking in young people: a systematic review. BMC Psychiatry.

[61] Rickwood DJ, Braithwaite VA (1994). Social-psychological factors affecting help-seeking for emotional problems. Soc Sci Med.

[62] Rickwood DJ, Deane FP, Wilson CJ (2007). When and how do young people seek professional help for mental health problems?. Med J Aust.

[63] Biddle L, Donovan J, Sharp D, Gunnell D (2007). Explaining non-help-seeking amongst young adults with mental distress: a dynamic interpretive model of illness behaviour. Sociol Health Illn.

[64] Marshall EG (2011). Do young adults have unmet healthcare needs?. J Adolesc Health.

[65] DeAndrea DC (2014). Advancing Warranting Theory. Commun Theor.

[66] Josephine K, Liat J, Richard FM (1997). APA PsycTests.

[67] Coralie JW, Frank PD, Joseph C, Debra R (2005). APA PsycTests.

[68] Shery M, Macneil C (2006). Peer support: what makes it unique. Int J Psychosoc Rehabil.

[69] Hinde N (2019). HuffPost UK.

[70] Tanner JL, Arnett JJ (2006). Emerging Adults in America: Coming of Age in the 21st Century.

[71] Riessman F (1965). The 'helper' therapy principle. Soc Work.

[72] Kelley C, Lee B, Wilcox L (2017). Self-tracking for mental wellness: understanding expert perspectives and student experiences. Proc SIGCHI Conf Hum Factor Comput Syst.

[73] Hinz LD (2009). Expressive Therapies Continuum: A Framework for Using Art in Therapy.

[74] Malchiodi CA (2013). Expressive Therapies.

[75] Murray EJ, Lamnin AD, Carver CS (1989). Emotional expression in written essays and psychotherapy. J Soc Clin Psychol.

[76] Isis P (2016). The Mindful Doodle Book.

[77] Fredrickson BL (2001). The role of positive emotions in positive psychology. The broaden-and-build theory of positive emotions. Am Psychol.

[78] Mahoney MJ, Snyder CR, Lopez SJ (2002). Constructivism and positive psychology. Handbook of Positive Psychology.

[79] Huh J, Ackerman MS (2012). Collaborative Help in Chronic Disease Management: Supporting Individualized Problems. Proceedings of the ACM 2012 conference on Computer Supported Cooperative Work.

